# Antiproliferative, Ultrastructural, and Physiological Effects of Amiodarone on Promastigote and Amastigote Forms of *Leishmania amazonensis*

**DOI:** 10.4061/2011/876021

**Published:** 2011-06-13

**Authors:** Sara Teixeira de Macedo-Silva, Thais Larissa Araújo de Oliveira Silva, Julio A. Urbina, Wanderley de Souza, Juliany Cola Fernandes Rodrigues

**Affiliations:** ^1^Laboratório de Ultraestrutura Celular Hertha Meyer, Instituto de Biofísica Carlos Chagas Filho, Universidade Federal do Rio de Janeiro, Avenida Carlos Chagas, 373, CCS, Ilha do Fundão, 21941-902 Rio de Janeiro, Brazil; ^2^Instituto Nacional de Ciência e Tecnologia de Biologia Estrutural e Bioimagem, Brazil; ^3^Instituto Venezolano de Investigaciones Cientificas, Caracas, Venezuela; ^4^Instituto Nacional de Metrologia, Normalização e Qualidade Industrial (Inmetro), 20261-232 Rio de Janeiro, Brazil; ^5^Pólo Avançado de Xerém, Universidade Federal do Rio de Janeiro, 25250-470 Rio de Janeiro, Brazil

## Abstract

Amiodarone (AMIO), the most frequently antiarrhythmic drug used for the symptomatic treatment of chronic Chagas' disease patients with cardiac compromise, has recently been shown to have also specific activity against fungi, *Trypanosoma cruzi* and *Leishmania*. In this work, we characterized the effects of AMIO on proliferation, mitochondrial physiology, and ultrastructure of *Leishmania amazonensis* promastigotes and intracellular amastigotes. The IC_50_ values were 4.21 and 0.46 *μ*M against promastigotes and intracellular amastigotes, respectively, indicating high selectivity for the clinically relevant stage. We also found that treatment with AMIO leads to a collapse of the mitochondrial membrane potential (ΔΨ*m*) and to an increase in the production of reactive oxygen species, in a dose-dependent manner. Fluorescence microscopy of cells labeled with JC-1, a marker for mitochondrial energization, and transmission electron microscopy confirmed severe alterations of the mitochondrion, including intense swelling and modification of its membranes. Other ultrastructural alterations included (1) presence of numerous lipid-storage bodies, (2) presence of large autophagosomes containing part of the cytoplasm and membrane profiles, sometimes in close association with the mitochondrion and endoplasmic reticulum, and (3) alterations in the chromatin condensation and plasma membrane integrity. Taken together, our results indicate that AMIO is a potent inhibitor of *L. amazonensis* growth, acting through irreversible alterations in the mitochondrial structure and function, which lead to cell death by necrosis, apoptosis and/or autophagy.

## 1. Introduction

Leishmaniasis is a parasitosis caused by different species of the *Leishmania* genus that affects about 12 million people around the world, with 90% of the cases reported in Afghanistan, Pakistan, Iran, Iraq, Syria, Saudi Arabia, India, Bangladesh, Nepal, Sudan, Algeria, Ethiopia, Brazil, Bolivia, Colombia, Ecuador, Peru, and Venezuela [1]. Approximately 21 species have been described to cause three different clinical manifestations: (1) cutaneous (CL), where the lesions are confined to the site of the inoculation by the sandfly; (2) mucocutaneous (MCL), which affects the mucosal tissues; (3) visceral (VL), where the parasites have a tropism for phagocytes mainly localized in the spleen and the liver. Visceral leishmaniasis is fatal if not treated, while some forms of cutaneous manifestations can cure spontaneously. According to WHO, around 70,000 deaths per year occur throughout the world [1]. In Brazil, *Leishmania amazonensis* is one of the species responsible for the cutaneous disease; however, in some individuals the immune system fails to mount an appropriate response against the parasite, leading to clinical manifestations of diffuse cutaneous leishmaniasis [2].

Pentavalent antimonials such as meglumine antimoniate (Glucantime) and sodium stibogluconate (Pentostan) have been employed as first-line treatment for many decades [3]. In the case of resistance to pentavalent antimonials, second-line treatments using amphotericin B or pentamidine have been successfully used [4]. For visceral leishmaniasis, miltefosine (Impavido) has been recently employed by oral route in India as a first-line treatment [5]. However, the drug is teratogenic and has a narrow chemotherapeutic window [6]. More recently, combination treatments are emerging as first-line treatments for visceral leishmaniasis [7]. Toxic side effects and increasing resistance limit most of the current specific treatments for leishmaniasis, indicating that there is an urgent need to develop new drugs that are efficacious, safe, and more accessible for the patient populations. 

Amiodarone (AMIO) is the antiarrhythmic class III drug most frequently used to treat arrhythmias in general as well as in patients with chronic Chagas' disease and cardiac compromise. The antiarrhythmic action in mammals has been well characterized and results from Ca^2+^ and K^+^channel inactivation, but it has recently been shown that the drug also has selective activity against parasitic protozoa such as *Trypanosoma cruzi *and *Leishmania mexicana* [8–10] as well as a broad-spectrum antifungal action [11, 12]. The mechanisms of action of AMIO reported in these different microorganisms involve the inhibition of sterol biosynthesis, disruption of mitochondrial membrane potential (*ΔΨm*), and Ca^2+^ homeostasis, as well as production of reactive oxygen species [8, 9, 11–13]. Apparently, these alterations in the mitochondrial metabolism trigger a sequence of cellular events leading to apoptosis-like cell death [13]. However, the action of AMIO on the mitochondrion of target cells is controversial, as some groups have implicated inhibition of mitochondrial respiration, mainly by the direct action of AMIO against complex I, II, and F0F1-ATP synthase [13] or by a rapid release of Ca^2+^ from the mitochondrial compartment due to a collapse of mitochondrial membrane potential (*ΔΨm*) (see [8, 9]), while others suggest that AMIO is able to protect mitochondrial function [14–17].

In this study, we investigated the antiproliferative, ultrastructural, and physiological effects of amiodarone on promastigote and intracellular amastigote forms of *Leishmania amazonensis*. The results indicated that AMIO acts mainly by altering the mitochondrial ultrastructure and physiology but other deleterious effects were also observed, including lipid accumulation, loss of the plasma membrane integrity, and presence of autophagic-like structures, suggesting different types of cell death involved in the mechanism of action of AMIO.

## 2. Material and Methods

### 2.1. Parasites

MHOM/BR/75/Josefa strain of *Leishmania amazonensis* isolated from a patient with diffuse cutaneous leishmaniasis by C. A. Cuba-Cuba (Universidade de Brasilia, Brazil) was used in the present study. It has been maintained by hamster footpad inoculation and, in the case of promastigotes, axenically cultured in Warren's medium (brain heart infusion plus hemin and folic acid) [18] supplemented with 10% fetal bovine serum at 25°C. Infective promastigotes of the Josefa strain were used to obtain intracellular amastigotes in macrophage cultures.

### 2.2. Drug

Amiodarone (AMIO), {(2-butyl-3-benzofuranyl)-[4-[2-(diethylamino)ethoxi]-3,5-diiodophenyl]methanone hydrochloride}, was purchased from Sigma, dissolved in dimethyl sulfoxide as a 100 mM stock, and stored at −20°C. 

### 2.3. In Vitro Antiproliferative Activities of Amiodarone

Growth experiments with promastigotes were initiated with 2.0 × 10^6^ parasites/mL, and AMIO was added at different concentrations from concentrated stock solutions in DMSO after 24 h of growth. Cell densities were evaluated daily in a Neubauer chamber during 72 h of growth. To evaluate the effects of the AMIO on the *L. amazonensis* intracellular amastigotes, peritoneal macrophages from CF1 mice were harvested by washing them with RPMI medium (Gibco) and plated in 24-well tissue culture chamber slides, allowing them to adhere to the slides for 24 h at 37°C in 5% CO_2_. Adherent macrophages were infected with metacyclic promastigotes at a macrophage-to-parasite ratio of 1 : 10 at 35°C for 2 h. After this time, noningested parasites were removed by washing and infected cultures were incubated for 24 h in RPMI (containing 10% of fetal bovine serum) without AMIO. Different concentrations of AMIO were added after 24 h of interaction, when the number of amastigotes per macrophage was in the range of two to four, and fresh medium with AMIO was added daily for 2 days. The cultures were fixed with 4% freshly prepared formaldehyde in phosphate buffer saline (PBS, pH 7.2) and stained with Giemsa for 15 min. The percentage of infected cells was determined by light microscopy. Association indexes (mean number of parasites internalized per cell, multiplied by the percentage of infected macrophages, and divided by the total number of macrophages) were determined and used as a parameter to calculate the intensity of infection in each condition used in this study. The 50% inhibitory concentrations (IC_50_s) were calculated with the SigmaPlot (version 10) program. The results are expressed as the means of three independent experiments.

### 2.4. Tests of Viability in Macrophages

To evaluate the cytotoxicity effects of AMIO against the host cell cultures, macrophages were incubated with different concentrations of AMIO for 48 h, and exclusion tests with 0.1% trypan blue were carried out for 5 min. The percentages of dead and alive cells were determined after counting of 400 macrophages in randomly selected fields by light microscopy. 

### 2.5. Estimation of Mitochondrial Transmembrane Electric Potential (ΔΨm)

*ΔΨm* of the control and AMIO-treated (6,10, and 15 *μ*M) promastigotes was investigated using the JC-1 fluorochrome, which is a lipophilic cationic mitochondrial vital dye that becomes concentrated in the mitochondria in response to *ΔΨm*. The dye exists as a monomer at low concentrations, where the emission is 530 nm (green fluorescence), but at higher concentrations it forms J-aggregates after accumulation in the mitochondrion, where the emission is 590 nm (red fluorescence). Thus, the fluorescence of JC-1 is considered an indicator of an energized mitochondrial state, and it has been used to measure the *ΔΨm* in *Leishmania* [19–21]. Control and AMIO-treated promastigotes after 48 h of treatment were harvested, washed in PBS, pH 7.2, added to a reaction medium containing 125 mM sucrose, 65 mM KCl, 10 mM HEPES/K^+^ pH 7.2, 2 mM Pi, 1 mM MgCl_2_, and 500 *μ*M EGTA, and counted in a Neubauer chamber. To evaluate the *ΔΨm* for each experimental condition, 2.0 × 10^7^ parasites were incubated in 10 *μ*g/mL JC-1 during 30 min, with readings made every 1 min using a Molecular Devices Microplate Reader (a spectrofluorometer SpectraMax M2/M2^e^). Cells were incubated in the presence of oligomycin (10 *μ*M), a F0F1-ATP synthase inhibitor, or FCCP (1 *μ*M), a mitochondrial protonophore, during the 30 min of experiment as positive controls of the depolarization of the mitochondrial membrane. FCCP at the concentration of 2 *μ*M was added at the end of all experiments to abolish *ΔΨm*. This allowed comparison of the magnitude of *ΔΨm* under the different experimental conditions. The relative *ΔΨm* value was obtained calculating the ratio between the reading at 590 nm and the reading at 530 nm (590 : 530 ratio). Control and AMIO-treated promastigotes were also observed under a Zeiss Axioplan epifluorescence microscope using different optical filter sets: (1) for J-aggregate alone, we used 546 nm band-pass filter for excitation, with a 580 nm beam splitter, and a 590 nm long-pass for emission; (2) for monomer and J-aggregate, together, we used a 450–490 nm for excitation, with a 510 nm beam splitter, and a 520 nm long pass for emission. Each experiment was repeated at least three times in triplicate, and the figures shown are representative of these experiments. 

### 2.6. Measurements of ROS Levels

Intracellular ROS level was measured in intact control and AMIO-treated promastigotes as described previously [19]. Briefly, cells (3.0 × 10^7^) were washed, resuspended in 500 *μ*L of PBS pH 7.2, and then incubated with the cell-permeable probe green H_2_DCFDA at a concentration of 10 *μ*g/mL for 1h at 25°C. After incubation, cells were analyzed at a Molecular Devices Microplate Reader (a spectrofluorometer SpectraMax M2/M2^e^, Molecular Devices) using a pair of 507 nm and 530 nm as emission and excitation wavelengths, respectively.

### 2.7. Evaluation of Membrane Integrity and Nile Red Accumulation

Control and AMIO-treated (6,10, and 15 *μ*M) promastigotes were harvested, washed in PBS, pH 7.2, and counted in a Neubauer chamber. After that, cells (5.0 × 10^6^) were incubated with 1 *μ*M SYTOX Blue and 10 *μ*g/mL Nile Red for 20 minutes. The experiments were made in triplicate, using a black 96-well plate. The final volume in each well was 200 *μ*L of cell suspension in PBS. The reading was done in a Molecular Devices Microplate Reader (a spectrofluorometer SpectraMax M2/M2^e^) according to the following wavelengths for excitation and emission, respectively: 444 and 560 nm for SYTOX Blue, and 485 and 538 nm for the Nile Red. After the readings, control and AMIO-treated cells were observed under a Zeiss Axioplan epifluorescence microscope equipped with optical filters to SYTOX Blue (the same of DAPI) and Nile Red (450–490 nm for excitation, and 528 nm for emission). Each experiment was repeated at least three times in triplicate, and the figures shown are representative of these experiments. 

### 2.8. Electron Microscopy

Control and AMIO-treated promastigotes were fixed for 3 h at 4°C in 2.5% glutaraldehyde (Sigma Chemical Co.) in 0.1M cacodylate buffer (pH 7.2). After fixation, cells were postfixed for 30 min in a solution containing 1% OsO_4_ and 1.25% potassium ferrocyanide in 0.1 M cacodylate buffer, washed in the same buffer, dehydrated in acetone, and embedded in EPON. Ultrathin sections were stained with uranyl acetate and lead citrate and observed in a Zeiss 900 electron microscope. 

## 3. Results and Discussion

### 3.1. Antiproliferative Effects

**Figure 1:**
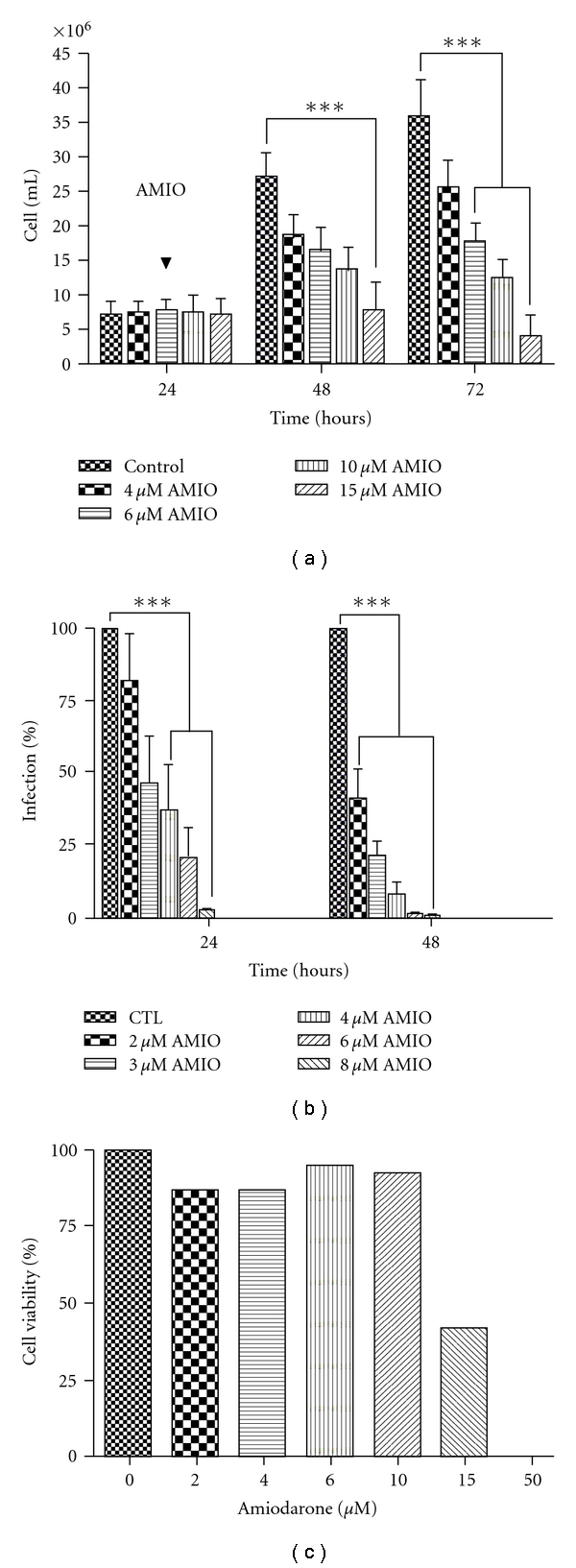
Antiproliferative and cytotoxic effects of amiodarone (AMIO) on *Leishmania amazonensis* promastigotes and intracellular amastigotes. (a) Treatment of promastigotes with different concentrations of AMIO for 48 h. (b) Effects of amiodarone on intracellular amastigote forms cultivated in macrophages. Murine host cells were cultivated for 24 h and infected with metacyclic promastigotes for 2 h. After 24 h of infection, AMIO at different concentrations was added to the cultures and treatment maintained for 48 h, with fresh medium with drug added every 24 h. (c) The cytotoxicity of AMIO was also evaluated in noninfected murine macrophages. After treatment with AMIO for 48 h, the macrophages were incubated with Trypan Blue and 400 cells were counted in randomly chosen fields under light microscopy. The experiments were carried out in triplicate, and the bars represent the standard deviation. Statistical analyses were obtained in Prism Software using 2-way ANOVA. The values of p were obtained comparing the control group with the treated groups: ***P < .001.

**Figure 2:**
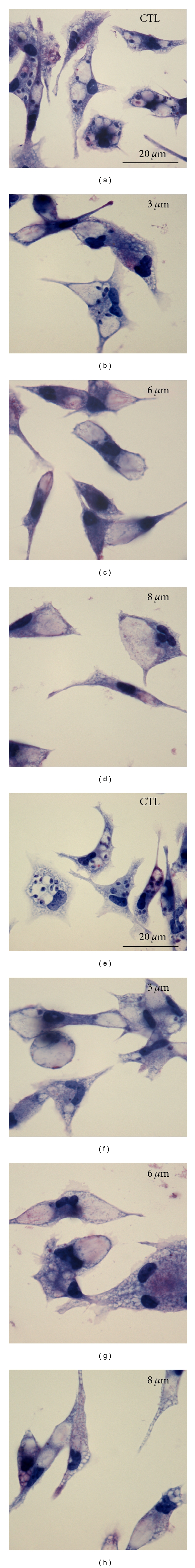
Light microscopy of intracellular amastigote forms of *Leishmania amazonensis* treated with different concentrations of amiodarone (AMIO). (a–d) After 24 h of treatment, it is possible to observe a significant reduction in the parasite number with 6 and 8 *μ*M AMIO (c, d). (e–h) After 48 h of treatment, a significant reduction was observed in the cultures treated with 3 *μ*M AMIO (f). Infection of macrophages was carried out as described in Materials and Methods and Figure 1. For all the images, the scale bars are similar to those observed in the panels (a) and (e). (a) Control parasites/24 h of treatment, which means 48 h of infection; (e) Control parasites/48 h of treatment, which means 72 h of infection.

*L. amazonensis* promastigotes were exposed to different concentrations of AMIO, and their proliferation was followed during 4 days.  Figure 1(a) shows a concentration-dependent inhibition of the growth induced by the treatment. The 50% inhibitory concentration (IC_50_) was 4.21 *μ*M after 48 h of treatment. Inhibition of around 100% was obtained with a concentration of 15 *μ*M AMIO, which also induced a delayed lytic effect. We also investigated the effects of AMIO on intracellular amastigotes, the clinically relevant form of the parasite. Macrophages were infected with metacyclic promastigotes and treated with different concentrations of the drug. The IC_50_ obtained was 0.46 *μ*M after 48 h of treatment, with a total elimination of amastigotes when macrophages were treated with 6 *μ*M for 48h (Figure 1(b)). Detailed inspection of treated cultures using light microscopy confirmed the potent antiproliferative effect against intracellular amastigotes, as no intact parasites were seen after treatment with concentrations ≥6 *μ*M of AMIO for 48 h (Figure 2).

Comparing our results with those published for *L. mexicana* [9], another species of the same *Leishmania* subgenus but that is not relevant for the epidemiology of leishmaniasis in Brazil, the IC_50_ values found in this study were higher for both stages (4.21 *μ*M for promastigotes and 0.46 *μ*M for intracellular amastigotes compared with 900 nM and 8 nM, respectively, for *L. mexicana*), indicating a lower susceptibility of our *L. amazonensis* strain to the drug. These results are consistent with the known fact that patients infected with *L. amazonensis* are less responsive to the available anti-*Leishmania* treatments [2]. It is interesting to point out that the clinically relevant intracellular amastigote form of the parasite had a ten-fold higher susceptibility to amiodarone than the extracellular promastigotes, a result similar to those obtained for *T. cruzi* [8] and *L. mexicana *[9]. 

 Using the cytotoxicity trypan blue test, viable cells were evaluated in macrophage cultures exposed to AMIO at concentrations varying between 2 and 50 *μ*M for 48 h (Figure 1(c)). The cytotoxicity concentration to reduce 50% (CC_50_) of viable macrophages was 12.9 *μ*M (Figure 1), giving a mean selectivity index of 28. Light microscopy showed that macrophages treated with 6 and 8 *μ*M AMIO did not present any significant alteration (Figure 2). In several mammalian cell lines, AMIO has been described to be very toxic [17, 22, 23]. However, the selectivity of AMIO for trypanosomatid parasites over mammalian cells and organisms has been confirmed in experimental models of Chagas' disease and leishmaniasis [8–10] and more recently in clinical studies [24, 25]. 

### 3.2. Effects of AMIO on the Neutral Lipid Accumulation and on the Fine Structure of *L. amazonensis* Promastigotes

In order to evaluate the effect of AMIO on the plasma membrane integrity and the presence of lipid-storage bodies in* L. amazonensis *promastigotes, we performed a quantitative analysis after simultaneous incubation of the cells with SYTOX Blue and Nile Red. The results indicated that at the higher concentration used (15 *μ*M AMIO), the plasma membrane permeability was significantly altered (data not shown). This effect was not observed at lower concentrations (6 and 10 *μ*M AMIO). 

**Figure 3:**
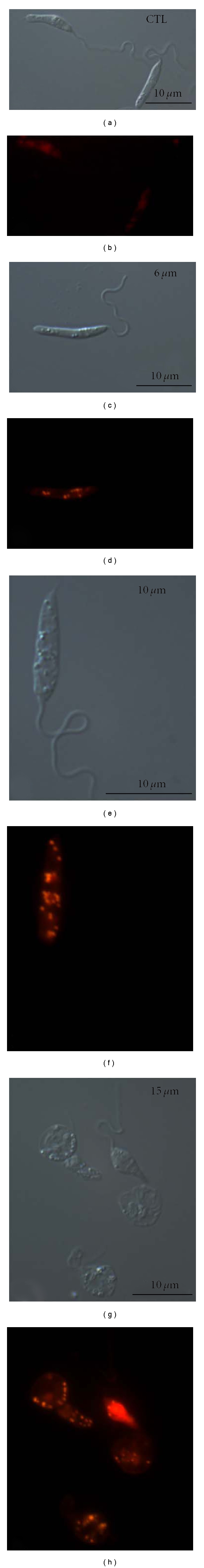
Differential interference contrast microscopy (DIC) and fluorescence microscopy with Nile Red of *Leishmania amazonensis* promastigotes untreated (a-b) and treated with different concentrations of AMIO (6, 10 and 15 *μ*M) for 48 h (c–h, resp.). In the treated promastigotes, images suggest an accumulation of lipid-storage bodies in the cytoplasm, which is concentration dependent. In (g), parasites treated with 15 *μ*M AMIO are completely modified. The conditions used to harvest the parasites, to incubate with Nile Red and to observe under the microscopy using the correct filters sets are described in Materials and Methods.

On the other hand, incubation with Nile Red revealed a concentration-dependent effect on neutral lipids' accumulation, which was quantified by fluorimeter (data not shown). The visualization of AMIO-treated promastigotes under fluorescence microscopy revealed the presence of many lipid bodies positive to Nile Red and randomly distributed throughout the cytoplasm (Figures 3(a)–3(h)). The fluorescence images indicate a concentration-dependent increase in the number of lipid bodies (Figures 3(d), 3(f), and 3(h)), while differential interference contrast microscopy (DIC) revealed an important alteration in the shape of promastigotes treated with 15 *μ*M AMIO, which appeared rounded and swollen with a clear evidence of loss of the cytoplasmatic content (Figure 3(g)), confirming the alteration on the plasma membrane integrity indicated by SYTOX Blue staining. These results are consistent with the reported effect of AMIO on the *de novo* sterol biosynthesis in *T. cruzi* [8] and *L. mexicana* [9]. Similar results were obtained with *L*. *amazonensis* treated with squalene synthase inhibitors [26].

**Figure 4:**
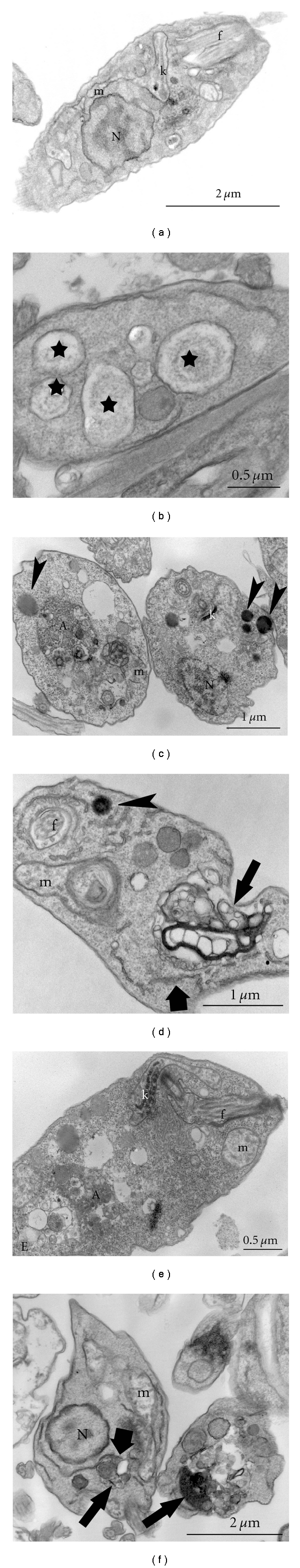
Different ultrastructural alterations on *Leishmania amazonensis* promastigotes induced by the treatment with amiodarone (AMIO). (a) Ultrathin section of *L. amazonensis* promastigotes without treatment, which presents a normal ultrastructure of organelles such as (mitochondrion) m, (kinetoplast) k, (nucleus) N and (flagellum) f. (b) Electron micrograph of *L. amazonensis* treated with 5 *μ*M AMIO for 48 h presenting many vacuoles similar to autophagosomes (stars). (c–e) After treatment with 15 *μ*M AMIO for 24 h, it is possible to observe the presence of large autophagosomes a associated with endoplasmic reticulum profiles (big arrow), lipid bodies (arrowheads), and alterations in the mitochondrion–kinetoplast complex and chromatin condensation. (f) Promastigotes treated with 20 *μ*M AMIO for 24 h presented drastic alterations and destruction of the cytoplasm, where it is possible to observe the presence of autophagosomes (arrows) sometimes associated with endoplasmic reticulum profile (big arrow). A: autophagosome; f: flagellum; k: kinetoplast; m: mitochondrion; N: nucleus.

We also evaluated the effect of AMIO on the fine structure of *L. amazonensis *promastigote.  Figure 4(a) shows a longitudinal section of a control promastigote presenting different organelles such as mitochondrion, nucleus, kinetoplast, and flagellum with normal ultrastructure. Parasites incubated in the presence of AMIO displayed significant morphological changes, which varied from discrete alterations to a total destruction of the parasite, depending on the drug concentration and length of incubation. The changes began to appear with 5 *μ*M AMIO after 48 h of incubation, when it was possible to observe the presence of some vacuoles similar to autophagosomes (Figure 4(b), stars). With just 24 h of treatment, but using higher concentrations (15 and 20 *μ*M), several alterations could be observed such as presence of lipid-storage bodies (arrowheads), large myelin-like figures (arrow) with presence of endoplasmic reticulum profiles (big arrow), and autophagic structures containing cellular debris (Figures 4(c)–4(f)). These structures could be related to a degradation of damaged organelle induced by the drug treatment. In addition, alterations in the mitochondrion, in the kinetoplast, and in the chromatin condensation can be observed in Figures 4(c)–4(d). We also observed the presence of several small vesicles inside the flagellar pocket (Figure 4(e)) and many cells swollen and completely destroyed after treatment with 15 and 20 *μ*M AMIO for 24 h (Figure 4(f)). All these alterations are characteristic of the three main types of cell death: apoptosis, necrosis, and autophagy (reviewed in [27]). 

Some of the lipid bodies appeared near the plasma membrane and the autophagic-like structures, which could be related, respectively, to alterations of the biophysical properties of the plasma membrane and degradation of abnormal lipids that accumulated as a consequence of the treatment. Thus, alterations of lipid composition resulting from treatment with AMIO could interfere with plasma membrane integrity, leading to cell death by necrosis.

### 3.3. Effects of AMIO on the Mitochondrial Physiology and Ultrastructure of *L. amazonensis* Promastigotes

**Figure 5:**
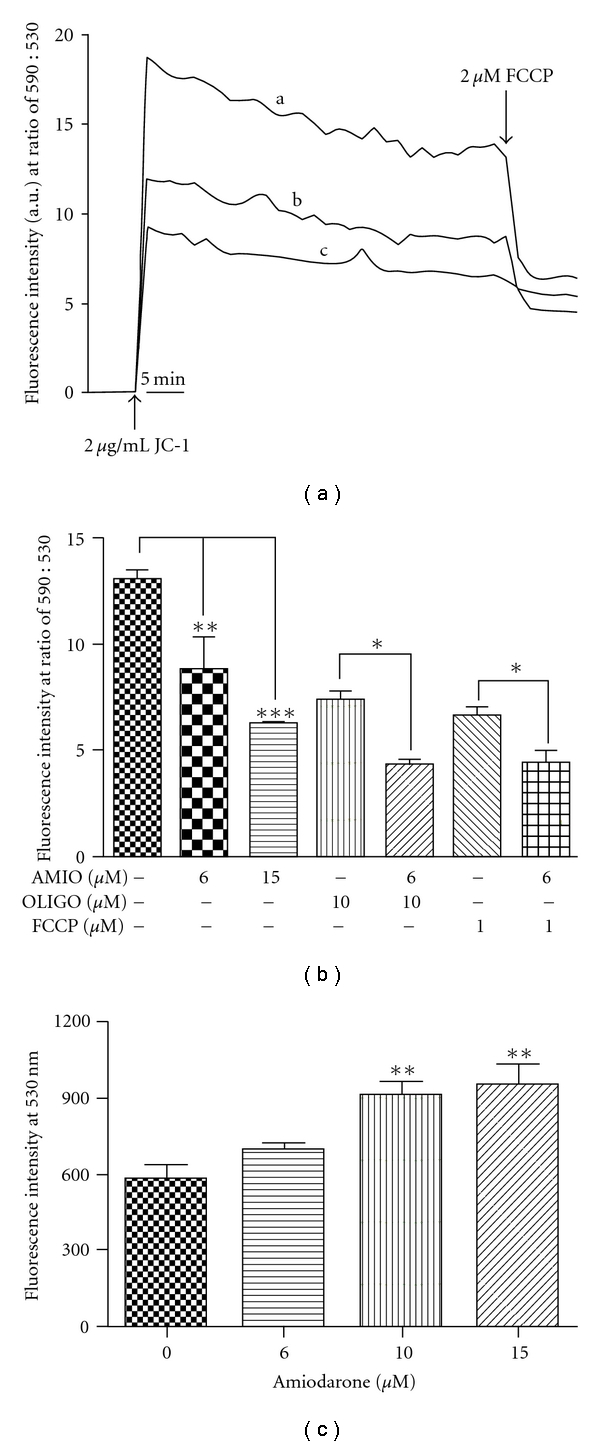
Effects of the AMIO on the mitochondrial function of *Leishmania amazonensis* promastigotes. (a) Values of *ΔΨm* were evaluated during 30 min, after which the uncoupler FCCP was added to collapse mitochondrial potential. Cells grown in the presence of two concentrations of amiodarone, 6 and 15 *μ*M, for 48 h (curves b and c, resp.) were observed. (b) Effect of oligomycin (10 *μ*M) and FCCP (1 *μ*M) in the *ΔΨm* was also evaluated. (c) Production of ROS was measured in the same cells. Control and treated cells were incubated with H_2_DCFDA, and the fluorescence intensity was measured in a Microplate Reader. Fluorescence intensity is expressed as arbitrary units (A.U.). The experiments were performed three times, each time in triplicate, and the figures shown are representative of these experiments.

We investigated the effect of AMIO on the mitochondrial function and ultrastructure using three criteria: mitochondrial transmembrane electric potential (*ΔΨm*) using JC-1 fluorochrome, production of reactive oxygen species (ROS) using a green H_2_DCFDA probe, and transmission electron microscopy. JC-1 is a cell-permeant cationic lipophilic fluorochrome that accumulates in the functional mitochondrion forming red-fluorescent J-aggregates. On the other hand, mitochondrial de-energization leads to an accumulation of green fluorescence monomers. Thus, the decrease in the red/green fluorescence intensity ratio indicates a collapse in the mitochondrial transmembrane potential. There are some advantages of using the JC-1: (1) it is not necessary to permeabilize the cells; (2) it is easy to quantify in a fluorimeter as well as to observe the process under a fluorescence microscopy. Promastigotes were treated with 0, 6, 10, and 15 *μ*M AMIO for 48 h prior to the analysis of the mitochondrial features. Incubation with JC-1 for 30 min indicated that cells treated with 6 and 15 *μ*M AMIO had a very significant reduction of *ΔΨm* (Figure 5(a), traces b and c) when compared with the control (untreated) parasites (Figure 5(a), trace a). The results indicated a marked reduction in the mitochondrial polarization with 6 *μ*M AMIO (Figure 5(a), trace b), and an almost total depolarization of the mitochondrial membrane potential with 15 *μ*M AMIO (Figure 5(a), trace c). After 30 min of JC-1 uptake, 2 *μ*M FCCP was added to fully collapse the *ΔΨm*: it can be seen that in control parasites the release of JC-1 was more prominent than in treated promastigotes. To compare these findings with other situations that can induce depolarization of the mitochondrion, control and treated parasites were incubated with two classical inhibitors of the mitochondrial function: olygomicin, which is an inhibitor of the F0F1-ATP synthase, and FCCP, a classical protonophore uncoupler that dissipates the mitochondrial electrochemical H+ gradient (Figure 5(b)). It can be seen that the reduction of *ΔΨm* found in cells grown in the presence of 6 and 15 *μ*M AMIO (Figure 5(a)) is similar to those obtained in control cells incubated with olygomicin or FCCP (Figure 5(b)). In addition, when these inhibitors were added in AMIO-treated promastigotes, the decrease of the *ΔΨm* was more evident.

We also investigated the effect of AMIO on the production of reactive oxygen species (ROS), as it is known that inhibition of oxidative phosphorylation induces an increase in the production of ROS. The results shown in Figure 5(c) indicate that cells grown in the presence of AMIO at the same concentrations used to evaluate the *ΔΨm*, showed very significant and concentration-dependent increase in the production of ROS. The effect was most evident with 10 and 15 *μ*M AMIO. 

**Figure 6:**
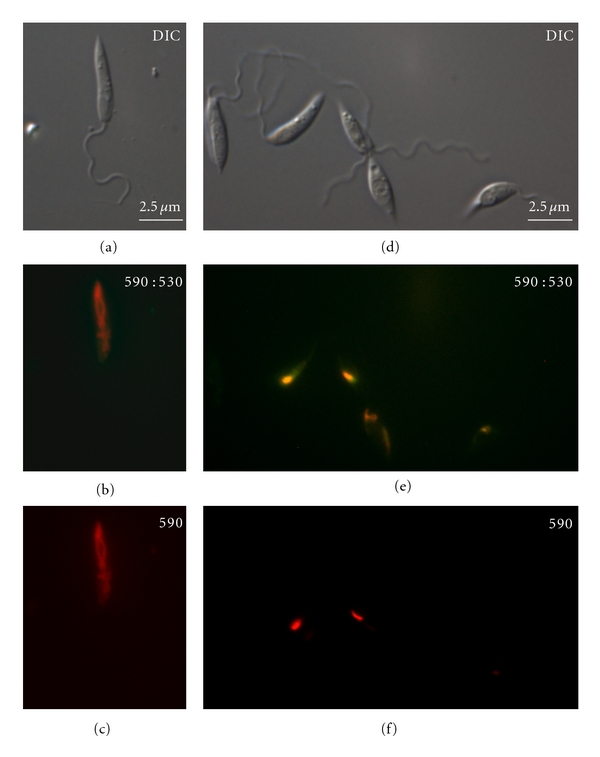
Differential interference contrast (DIC) microscopy and fluorescence microscopy with JC-1 of *Leishmania amazonensis* promastigotes untreated (a–c) and treated with 15 *μ*M AMIO for 48 h (d–f). In panels (b) and (c), the accumulation of aggregated-JC1 is observed in the whole extension of the control mitochondrion. In cells treated with 15 *μ*M AMIO (e-f), the accumulation of J-aggregates occurs in some portions of the mitochondrion, indicating a partial dissipation of the *ΔΨm*. Panels (b) and (e) show an image of the monomers and J-aggregates together, while panels (c) and (f) show the image of only J-aggregates.

Alterations of the mitochondrion were also investigated using fluorescence and transmission electron microscopy, confirming the results obtained at the fluorimeter. Visualization of control and AMIO-treated promastigotes incubated with JC-1 under fluorescence microscopy revealed that cells grown with 15 *μ*M AMIO for 48 h showed reduced accumulation of the fluorochrome, restricted to the kinetoplast region of the single giant mitochondrion (Figures 6(e) and 6(f)), indicating a loss of only *ΔΨm* in most parts of the organelle. In contrast, in control promastigotes, JC-1 accumulated in the whole extension of the ramified mitochondrion (Figures 6(b) and 6(c)). Images (b) and (e) show the fluorescence for monomers and J-aggregates, together, while the images (c) and (f) show the fluorescence for J-aggregates alone. 

**Figure 7:**
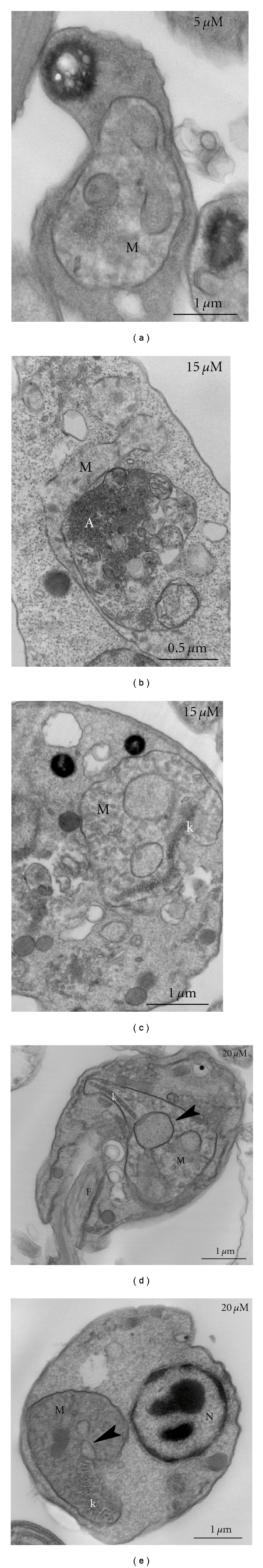
Ultrathin sections of *L. amazonensis* promastigotes treated with different concentrations of AMIO showing several alterations in the mitochondrial ultrastructure such as marked swelling (a, c, d, and e) with loss of the matrix content (a), alterations in the mitochondrial membrane (arrowheads), and presence of autophagic structures near a modified mitochondrion (b). A: autophagic structure; F: flagellum; k: kinetoplast; M: mitochondrion, N: nucleus.

Finally, ultrastructural alterations on the mitochondrion were also investigated: in Figure 7 the occurrence of dramatic modifications in different aspects of the organelle can be seen. It is evident that long-term incubation with AMIO induced mitochondrial swelling at all the concentrations tested (Figures 7(a), 7(c), 7(d) and 7(e)), which is followed by alterations in the mitochondrial membranes (Figures 7(d) and 7(e), arrowheads), appearance of circular cristae (Figure 7(a) and 7(c)), and loss of the mitochondrial matrix content (Figures 7(a) and 7(d)). In addition, we also observed an important interaction between the mitochondrion and structures similar to autophagic vacuoles (Figure 7(c)). In this same figure, alterations in the kinetoplast structure are also evident. Ultrastructural alterations on the mitochondrion also predominate in *T. cruzi* and *Leishmania* cells treated with sterol biosynthesis inhibitors, including quinuclidine derivatives, azoles and azasterols, indicating that this organelle is an important target for compounds that interfere with sterol and lipid composition [27–35].

The dose-dependent reduction of *ΔΨm* induced by amiodarone was correlated with the increase in the production of reactive oxygen species and mitochondrial ultrastructural alterations detected with transmission electron microscopy. Two possible explanations for these combined alterations are: (1) Calcium release from the mitochondrion, which would result from a direct action of AMIO on the organelle and lead to apoptotic cell death [36]; and, (2) Sterol biosynthesis inhibition, as observed in fungi [37] and trypanosomatids [8, 9], leading to an important alteration in the lipid composition of the mitochondrial membranes that should modify their biophysical properties [28, 38, 39], and loss of mitochondrial function. 

## 4. Conclusion

In conclusion, the results of this work support the potential usefulness of AMIO as a chemotherapeutic agent against *Leishmania amazonensis*. Such activity is mainly mediated by profound and selective effects on the ultrastructure and physiology of the parasite mitochondrion, which culminate in cell death by necrosis, apoptosis, or autophagy. More studies are necessary to better characterize the different types of cell death associated with the mechanisms of action of AMIO and the activity of the drug in murine models of cutaneous leishmaniasis by *L. amazonensis*.
